# Supplementary material for: Projection of Expression Profiles to Transcription Factor Activity Space Provides Added Information
Source: Genes (Basel). 2022 Oct 8;13(10):1819. doi: 10.3390/genes13101819 (PMC9601664; doi:10.3390/genes13101819)
Supplement: Supplementary file 1 [file genes-13-01819-s001.zip › genes-1818854-supplementary.pdf]

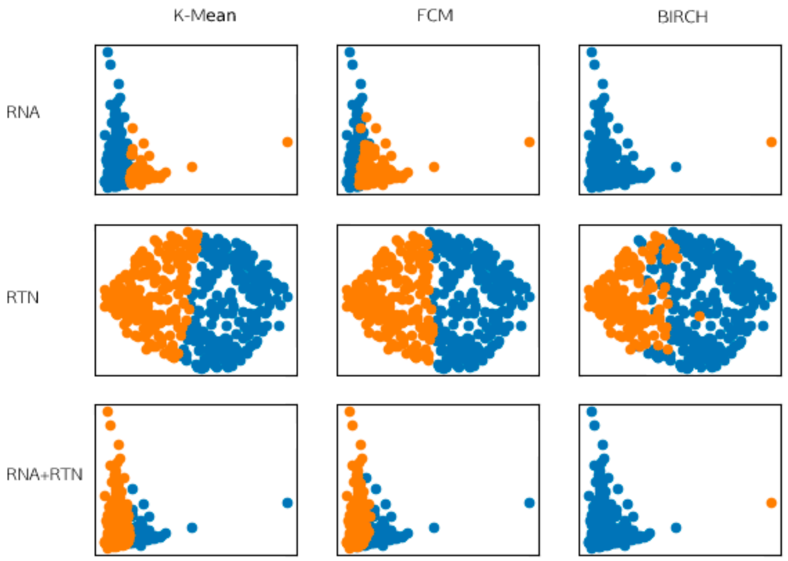

Figure S1. Clustering visualization. Each point represents the tumor of one patient; points are colored by grouping, shown with the first (X-axis) and the second (Y-axis) dimensions of PCA.

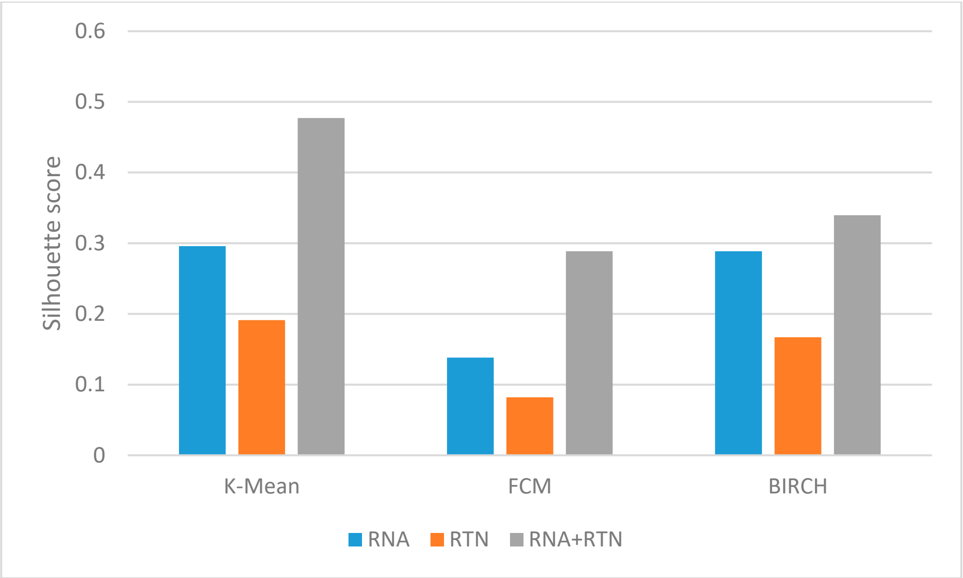

Figure S2. Silhouette Coefficient score of the different datasets on the different clustering method on population 2 (OHSU dataset) after feature selection.
